# Protein Supplementation Enhances the Effects of Intermittent Loading on Skeletal Muscles by Activating the mTORC1 Signaling Pathway in a Rat Model of Disuse Atrophy

**DOI:** 10.3390/nu12092729

**Published:** 2020-09-07

**Authors:** Sho Miyatake, Kazuo Hino, Yuko Natsui, Goro Ebisu, Satoshi Fujita

**Affiliations:** 1 OS-1 Division, Medical Foods Research Institute, Otsuka Pharmaceutical Factory, Inc. 115 Kuguhara, Tateiwa, Muya-cho, Naruto, Tokushima 772-8601, Japan; Miyatake.Sho@otsuka.jp (S.M.); hinoka@otsuka.jp (K.H.); Natsui.Yuko@otsuka.jp (Y.N.); ebisug@otsuka.jp (G.E.); 2 Faculty of Sport and Health Science, Ritsumeikan University, 1-1-1, Nojihigashi, Kusatsu, Shiga 525-8577, Japan

**Keywords:** hind limb unloading, intermittent loading, protein, skeletal muscle

## Abstract

Inactivity leads to skeletal muscle atrophy, whereas intermittent loading (IL) during hind limb unloading (HU) attenuates muscle atrophy. However, the combined effects of IL and protein supplementation on disuse muscle atrophy are unclear. Therefore, we investigated the effects of IL and a high-protein oral nutritional supplement (HP) during HU on skeletal muscle mass and protein synthesis/breakdown. Male F344 rats were assigned to the control (CON), 14-day HU (HU), IL during HU (HU + IL), and IL during HU followed by HP administration (2.6 g protein/kg/day; HU + IL + HP) groups. Soleus and gastrocnemius muscles were sampled 30 min after the last IL and HP supplementation. HU decreased relative soleus and gastrocnemius muscle masses. Relative muscle masses and p70 ribosomal protein S6 kinase/ribosomal protein S6 phosphorylation in soleus and gastrocnemius muscles were higher in the HU + IL group than the HU group and further higher in the HU + IL + HP group than the HU + IL group in gastrocnemius muscle. Therefore, protein administration plus IL effectively prevented skeletal muscle atrophy induced by disuse, potentially via enhanced activation of targets downstream of mammalian target of rapamycin complex 1 (mTORC1) signaling pathway.

## 1. Introduction

Skeletal muscle mass is determined by the balance between protein synthesis and breakdown [1]. Strategies that increase muscle protein synthesis and reduce protein breakdown are important for maintaining and increasing skeletal muscle mass. Muscle protein synthesis is enhanced by resistance exercise and amino acids derived from dietary protein [2,3]. Essential amino acids (EAAs) stimulate muscle protein synthesis, and branched-chain amino acids (BCAAs), particularly leucine, exhibit strong anabolic effects [4,5].

Skeletal muscle atrophy is induced by aging, starvation, inactivity, cancer cachexia, diabetes, chronic kidney disease, and many other pathologies [6,7,8,9]. Disuse-muscle atrophy is the loss of skeletal muscle under conditions such as cast immobilization, prolonged bed rest, and microgravity [10]. To prevent disuse muscle atrophy, it is crucial to establish suitable and effective treatments with exercise and nutritional intervention.

The mammalian target of rapamycin complex 1 (mTORC1) signaling pathway is a key regulator related to muscle protein synthesis [11]. Insulin-like growth factor-1 binds to its receptor and activates phosphoinositide 3-kinase and Akt, resulting in activation of the mTORC1 signaling pathway [11]. p70 ribosomal protein S6 kinase (p70S6K), a signaling protein that functions downstream of mTORC1, activates ribosomal protein S6 (rpS6) via phosphorylation and enhances protein synthesis [12]. Previous reports have shown that exercise increases muscle protein synthesis by activating mTORC1 via Akt, whereas BCAAs increase muscle protein synthesis by directly activating mTORC1 [12,13].

Dupont et al. reported that hind limb unloading (HU)-induced muscle atrophy reduced Akt, mTORC1, and p70S6K phosphorylation in rat soleus (SOL) muscles [14]. Thus, attenuation of the mTORC1 signaling pathway reduces muscle protein synthesis in disuse muscle atrophy [15,16]. Previously, intermittent loading (IL) for 4 h per day was shown to increase the cross-sectional area of rat SOL muscles by activating the mTORC1 signaling pathway in disuse muscle atrophy induced by 7 days of HU [17]. However, the combined effects of protein supplementation and IL on disuse muscle atrophy have not been sufficiently investigated.

The ubiquitin-proteasome and autophagy-lysosome systems play important roles in skeletal muscle protein breakdown [18]. In disuse muscle atrophy, inhibition of the ubiquitin-proteasome system attenuates skeletal muscle atrophy, implying that this system is the major protein-breakdown system [19,20,21]. The muscle-specific E3 ubiquitin ligases atrogin-1 (also known as muscle atrophy F-box) and muscle ring finger 1 (MuRF1) are upregulated in various models of skeletal muscle atrophy [22,23]. Therefore, the expression levels of these genes are useful markers of skeletal muscle atrophy in the ubiquitin-proteasome system. In a rat model of HU-induced atrophy, IL inhibited atrogin-1 and MuRF1 gene expression in rat SOL muscles [17]. In addition, chronic oral administration of BCAAs was reported to increase the skeletal muscle cross-sectional area and decrease skeletal muscle atrogin-1 and MuRF1 expression in rat model of muscle atrophy induced by 14 days of HU [24,25]. However, the combined effects of long-term IL and protein supplementation on muscle protein synthesis and breakdown in disuse muscle atrophy have not been investigated.

Autophagy-related gene expression in skeletal muscle was increased in a model of muscle atrophy induced by fasting or denervation [26], suggesting that the autophagy-lysosome system is also important in skeletal muscle atrophy. The levels of LC3-II (phosphatidylethanolamine-conjugated LC3-I), the first mammalian protein identified that specifically associates with autophagosome membranes, are positively correlated with autophagosome formation, and this protein is recognized as a marker of autophagosome induction [27]. Unc-51-like autophagy-activating kinase 1 (ULK1) is a component of the ULK1 protein kinase complex, and phosphorylation of ULK1 at Ser757 is associated with autophagy inhibition [28,29]. The autophagy-lysosome system is activated in rat SOL and gastrocnemius (GS) muscles after 14 days of HU [30]. In contrast, no significant changes are observed in GS and quadriceps femoris muscles [31]. Thus, the mechanisms through which the autophagy-lysosome system participates in disuse muscle atrophy are unclear.

The central hypothesis of this study was that a combination of IL and protein supplementation could effectively inhibit disuse muscle atrophy. This hypothesis was validated using a rat model of HU-induced muscle atrophy. This animal model was established to investigate disuse muscle atrophy and has been used in studies primarily focused on SOL muscles demonstrating marked atrophy. Using this model, we assessed the effects of IL and oral administration of a high-protein liquid nutritional supplement (HP) on skeletal muscle mass, the expression levels of proteins related to muscle protein synthesis (mTORC1 signaling pathway), and protein breakdown in SOL and GS muscles via the ubiquitin-proteasome and autophagy-lysosome systems.

## 2. Materials and Methods

### 2.1. Animal and Experimental Protocols

This study was conducted in accordance with the Institutional Committee on Care and Use of Laboratory Animals of Otsuka Pharmaceutical Factory, Inc. (Tokushima, Japan) and was approved by the Otsuka Pharmaceutical Animal Experiment Committee (approval number: OPFCAE-18-398; approval date: 7 December 2018). Seven-week-old male F344/DuCrlCrlj rats were purchased from Charles River Japan (Yokohama, Japan). The rats were housed in a room at 23 ± 3 °C with 12-h light/dark cycle (light: 7:00 a.m.–7:00 p.m.) and 55% ± 15% humidity. After a 14-day acclimation period, the rats were divided into the following four groups: control (CON, *n* = 6), HU (*n* = 9), IL during HU (HU + IL, *n* = 10), and IL during HU followed by HP administration (HU + IL + HP, *n* = 10). Rats in the HU, HU + IL, and HU + IL + HP groups were subjected to HU for 14 days. During the experimental period, the CON and experimental rats were pair-fed an AIN-93G-based 10% casein diet (Oriental Yeast, Osaka, Japan; 9 g/day, protein: 4.1 g/kg/day) and provided water ad libitum. Two times per day (9:00 a.m. and 5:00 p.m.), the rats in the HU + IL + HP group were orally administered HP (HINEX REHADAYS; Otsuka Pharmaceutical Factory, Inc., energy: 38.4 kcal/kg/day; protein: 2.6 g/kg/day; fat: 0.5 g/kg/day; carbohydrates: 5.8 g/kg/day). HP contains 4.8 g/100 mL milk protein and 2.0 g/100 mL whey protein as its protein source and supplemented with 1.1 g/100 mL leucine and 0.8 g/100 mL citrulline as free amino acids. All rats in the other groups were orally administered a control, liquid oral-nutritional supplement (energy: 38.4 kcal/kg/day; protein: 3.2 g/kg/day; fat: 0.5 g/kg/day; carbohydrate: 5.6 g/kg/day), in which all of the proteins/amino acids in the HP were replaced with non-EAAs (alanine, aspartic acid, glutamic acid, glycine, proline, and serine in equimolar ratios), using the same dose and time as the HU + IL + HP group. Food intake was measured at 4:00 p.m. daily with an electronic balance. Following 15 h of fasting, blood and SOL and GS muscle samples were obtained under isoflurane anesthesia, 30 min after the last round of IL and oral administration. After weighing the tissues, they were rapidly frozen in liquid nitrogen and stored at −80 °C until analysis.

### 2.2. HU and IL

HU was performed as previously described by Morey et al. [32]. The rats were suspended by their tails using adhesive tape to prevent the hind limbs from contacting the cage floor during the experimental period. The rats were suspended by the tail at an angle of 30° from the head down and were allowed free access to water and food. IL was carried out for 1 h per day from 4:00–5:00 p.m. by releasing the rats from HU and allowing them to engage in normal cage activity.

### 2.3. Blood Analysis

Blood samples were collected from the abdominal aorta and drawn into blood collection tubes containing ethylenediaminetetraacetic acid disodium salt (Na_2_EDTA) and then immediately cooled on ice. Blood samples were centrifuged for 10 min at 4 °C and 3000 rpm, after which the plasma supernatants were collected. All samples were stored at −80 °C. The amino acid concentrations were determined by liquid chromatography-mass spectrometry (LC-MS; analysis commissioned to SRL, Inc. Tokyo, Japan).

### 2.4. Western Blot Analysis

Western blotting was performed as previously described [33], with minor modifications. Briefly, skeletal muscle samples were homogenized in RIPA buffer (20 mM Tris-HCl [pH 7.5], 150 mM NaCl, 1 mM Na_2_EDTA, 1 mM EGTA, 1% NP-40, 1% sodium deoxycholate, 2.5 mM sodium pyrophosphate, 1 mM β-glycerophosphate, 1 mM Na_3_VO_4_, 1 μg/mL leupeptin; Cell Signaling Technology, Danvers, MA, USA). Appropriate amounts of protease inhibitor cocktail (cOmplete Mini, EDTA-free; Roche, Mannheim, Germany) and phosphatase-inhibitor cocktail (PhosSTOP; Roche, Mannheim, Germany) were added to the RIPA buffer in advance. The homogenates were centrifuged at 15,000 rpm for 10 min at 4 °C. The supernatants were collected, and the protein concentrations were determined using a Protein Assay Rapid Kit wako II (Wako, Osaka, Japan). The samples were diluted with 3× sample buffer (5.0% [*v*/*v*] β-mercaptoethanol, 187.5 mM Tris-HCl [pH 6.8, 25 °C], 6% [*w*/*v*] sodium dodecyl sulfate [SDS], 30% glycerol, and 0.03% [w/v] bromophenol blue; Cell Signaling Technology) and boiled for 7 min at 97 °C. For each sample, 30 µg of protein was separated by electrophoresis using 5–20% gradient SDS polyacrylamide gels, and the proteins were then transferred to polyvinylidene difluoride membranes. Subsequently, the membranes were washed with Tris-buffered saline containing 0.05% Tween 20 (TBS-T, pH 7.6) for 1 h at 20–23 °C. The membranes were blocked with TBS-T containing bovine serum albumin for 1 h at 20–23 °C. The membranes were then washed with TBS-T and incubated overnight at 4 °C with primary antibodies (Cell Signaling Technology, Danvers, MA, USA) against phospho-Akt (Ser473) (catalog number 9271), total Akt (catalog number 9272), phospho-p70 S6 kinase (Thr389) (catalog number 9205), total p70 S6 Kinase (catalog number 2708), phospho-S6 ribosomal protein (Ser240/244) (catalog number 2215), total S6 ribosomal protein (catalog number 2217), LC3B (catalog number 2775), phospho-ULK1 (Ser757) (catalog number 14202), and total ULK1 (catalog number 8054). The membranes were then washed again in TBS-T and incubated with appropriate secondary antibodies for 1 h at 20–23 °C. Protein bands were detected using a chemiluminescent reagent (Luminata Western HRP substrate; Merck, Darmstadt, Germany). Protein bands were scanned using a chemiluminescence detector (Molecular Imager VersaDoc MP 500; Bio-Rad Laboratories, Hercules, CA, USA), and band intensities were quantified using ImageJ software, version 1.50i (National Institutes of Health, Bethesda, MD, USA). Protein-phosphorylation levels were normalized to the total levels of the corresponding protein. The autophagy-lysosome system was assessed by evaluating the LC3B II: LC3B I (LC3B II/I) ratio.

### 2.5. Quantitative Polymerase Chain Reaction (qPCR) Analysis

Total RNA was extracted from 20 mg of each skeletal muscle using RNeasy Fibrous Tissue Mini Kit (Qiagen, Hilden, Germany). The RNA concentration was determined using a NanoDrop 2000c UV-Vis spectrophotometer (Thermo Fisher Scientific, MA, USA). The mRNA expression levels of atrogin-1 (Rn00591730_m1) and MuRF-1 (Rn00590197_m1) were quantified using TaqMan Gene Expression Assays (Applied Biosystems, Waltham, MA, USA). The expression of 18S ribosomal RNA (TaqMan assay Rn03928990_g1) was detected for normalization purposes. qPCR was performed using a TaqMan RNA-to-CT 1-Step Kit (Thermo Fisher Scientific) in a 96-well reaction plate. Each well contained 60 ng RNA, 10 µL TaqMan RT-PCR mix, 0.5 µL TaqMan RT enzyme mix, 5.5 μL RNase-free water, and 1 µL of a TaqMan Gene Expression assay in a reaction volume of 10 μL. A 7500 Fast Real-Time PCR System (Thermo Fisher Scientific) was used to perform qPCR using the following thermocycling parameters: 48 °C for 15 min, 95 °C for 10 min, followed by 40 cycles of 95 °C for 15 s and 60 °C for 1 min. Relative gene expression levels were calculated using the 2^−ΔΔ*C*t^ method [34].

### 2.6. Statistical Analysis

Data are presented as the mean ± standard deviation (SD). Shapiro-Wilk normality test was performed to confirm normality. Differences in body weight, caloric intake, and skeletal muscle mass were assessed by one-way analysis of variance (ANOVA), followed by an unpaired Student’s t-test or Tukey’s multiple-comparison test to determine specific group differences. Differences in plasma amino acid concentrations, as well as mRNA and protein expression levels were assessed by a nonparametric ANOVA (Kruskal–Wallis test) followed by Mann–Whitney U tests or Steel–Dwass tests. Statistics were processed with EXSUS software (version 7.7, CAC Croit Corporation, Tokyo, Japan). Results with *p* values of less than 0.05 were considered statistically significant.

## 3. Results

### 3.1. Body Weight, Caloric Intake, and Skeletal Muscle Mass

The body weights, caloric intake, and skeletal muscle masses of the rats in each group are shown in Table 1. Body weight was significantly lower following 14 days of HU (*p* < 0.05). Although the mean caloric intake was significantly lower in the HU group than in the CON group (*p* < 0.05), no significant differences were observed among the HU, HU + IL, and HU + IL + HP groups.

The SOL muscle mass: body weight ratio in the HU group decreased to 58.5% of that in the CON group (*p* < 0.05). The relative SOL muscle mass was significantly higher in the HU + IL group than in the HU group (*p* < 0.05), whereas no difference was observed between the HU + IL and HU + IL + HP groups.

The GS muscle mass: body weight ratio in the HU group decreased to 89.4% of that in the CON group (*p* < 0.05). In addition, relative GS mass was significantly higher in the HU + IL group than in the HU group (*p* < 0.05), and the HU + IL + HP group demonstrated a significantly higher GS mass than the HU + IL group (*p* < 0.05).

### 3.2. Plasma Amino Acid Concentrations

Measured plasma amino acid concentrations are shown in Table 2. Blood samples were collected 30 min after the last oral administration, and plasma amino acid concentrations were determined by LC-MS analysis. Plasma EAA concentrations were significantly higher (*p* < 0.05) in the HU + IL + HP group than in the HU and HU + IL groups. The plasma concentrations of BCAAs and leucine were also significantly higher in the HU + IL + HP group than in the HU and HU + IL groups (*p* < 0.05).

### 3.3. Phosphorylation of Akt, p70S6K, and rpS6

**Figure 1:**
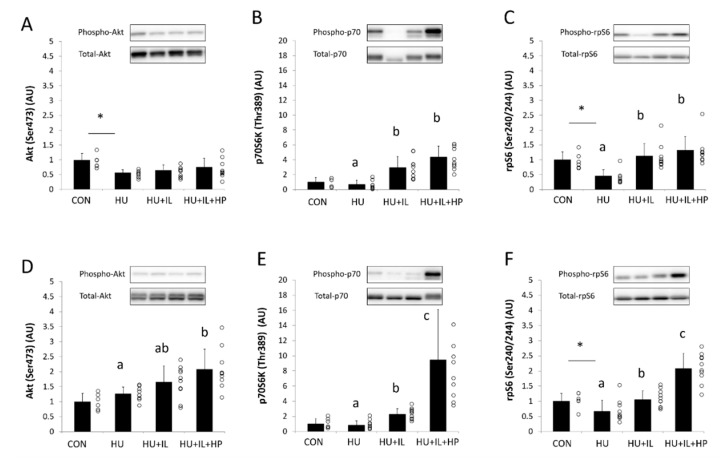
Effects of intermittent loading (IL) and high-protein oral nutritional supplement (HP) administration on the expression levels of phosphorylated Akt (Ser473), p70S6K (Thr389), and rpS6 (Ser240/244) in (**A**–**C**) soleus muscle and (**D**–**F**) gastrocnemius muscle. Four groups of rats were assigned: control (CON), hind limb unloading (HU), IL during HU (HU + IL), and IL during HU followed by HP administration (HU + IL + HP). The data are shown as the mean ± SD (*n* = 6–10). * *p* < 0.05, compared with the CON group. a, b, and c: different letters above the bars indicate significant differences among the HU, HU + IL, and HU + IL + HP groups as determined using multiple comparison tests (*p* < 0.05). Circles represent individual data points in each group. AU: arbitrary unit.

The phosphorylation levels of Akt at Ser473, p70S6K at Thr389, and rpS6 at Ser240/244 are shown in Figure 1. In SOL muscles, significantly higher p70S6K and rpS6 phosphorylation occurred in the HU + IL and HU + IL + HP groups than in the HU group (*p* < 0.05, Figure 1B,C). No significant changes were observed in the phosphorylation of Akt, p70S6K, and rpS6 in SOL muscles between the HU + IL and HU + IL + HP groups. In GS muscles, Akt phosphorylation was significantly higher in the HU + IL + HP group than in the HU group (*p* < 0.05, Figure 1D). In addition, the phosphorylation of p70S6K and rpS6 in GS muscles was significantly higher in the HU + IL group than in the HU group (*p* < 0.05, Figure 1E,F) and higher in the HU + IL + HP group than in the HU + IL group (*p* < 0.05).

### 3.4. mRNA Expression of Atrogin-1 and MuRF1

**Figure 2:**
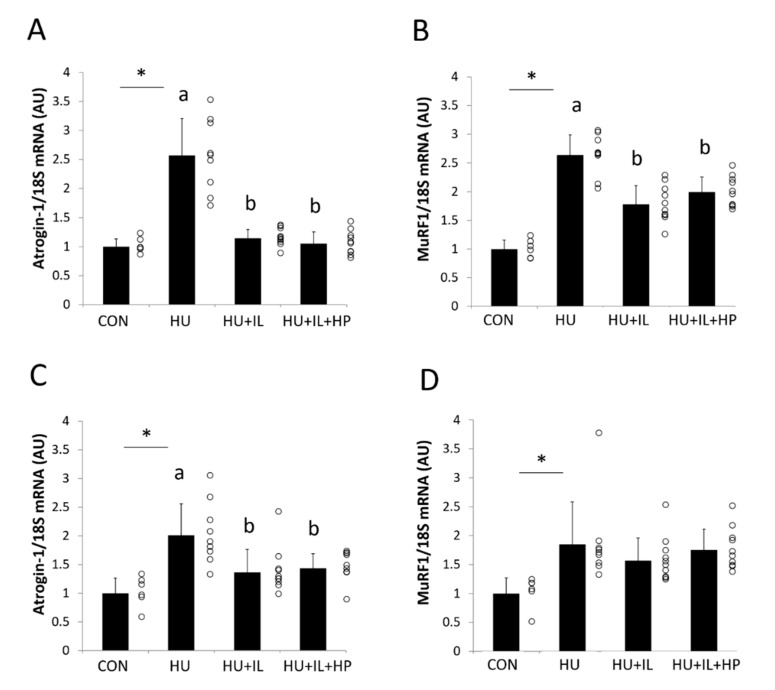
Effects of intermittent loading (IL) and high-protein oral nutritional supplement (HP) administration on the mRNA expression levels of atrogin-1 and MuRF1 in (**A**,**B**) soleus muscle and (**C**,**D**) gastrocnemius muscle. Four groups of rats were assigned: control (CON), hind limb unloading (HU), IL during HU (HU + IL), and IL during HU followed by HP administration (HU + IL + HP). The data are shown as the mean ± SD (*n* = 6–10). * *p* < 0.05, compared with the CON group. a and b: different letters above the bars indicate significant differences among the HU, HU + IL, and HU + IL + HP groups as determined using multiple comparison tests (*p* < 0.05). Circles represent individual data points in each group. AU: arbitrary unit.

Figure 2 shows the gene expression levels of molecular markers (atrogin-1 and MuRF1) of the ubiquitin-proteasome system. Atrogin-1 and MuRF1 mRNA levels in SOL muscles were significantly higher in the HU group than in the CON group (*p* < 0.05, Figure 2A,B) and were significantly lower in the HU + IL and HU + IL + HP groups than in the HU group (*p* < 0.05). Atrogin-1 and MuRF1 mRNA levels in GS muscles were also significantly higher in the HU group than in the CON group (*p* < 0.05, Figure 2C,D), and atrogin-1 mRNA expression was significantly lower in the HU + IL and HU + IL + HP groups than in the HU group (*p* < 0.05). The atrogin-1 and MuRF1 mRNA expression levels did not significantly differ between the HU + IL and HU + IL + HP groups, in both SOL and GS muscles.

### 3.5. Autophagy-Related Proteins LC3-II and ULK1

**Figure 3:**
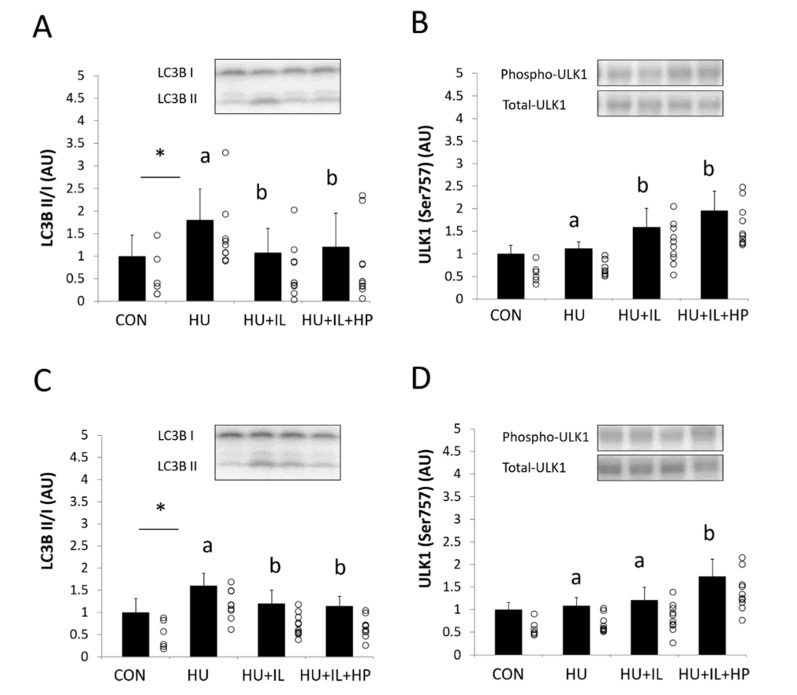
Effects of intermittent loading (IL) and high-protein oral nutritional supplement (HP) administration on the LC3B II/I ratio and levels of phosphorylated ULK1 in (**A**,**B**) soleus muscles and (**C**,**D**) gastrocnemius muscles. Four groups of rats were assigned: control (CON), HU, IL during HU (HU + IL), and IL during HU followed by HP administration (HU + IL + HP). The data are shown as the mean ± SD (*n* = 6–10). * *p* < 0.05: compared with the CON group. a and b: different letters above the bars indicate significant difference among the HU, HU + IL, and HU + IL + HP groups as determined using multiple comparison tests (*p* < 0.05). Circles represent individual data points in each group. AU: arbitrary unit.

Figure 3 shows the LC3B II/I ratio and the phosphorylation levels of ULK1 (Ser757), which are related to autophagy regulation. The LC3B II/I ratio in SOL and GS muscles was significantly higher in the HU group than in the CON group (*p* < 0.05, Figure 3A,C). Although the LC3B II/I ratio in SOL and GS muscles was significantly lower in the HU + IL and HU + IL + HP groups than in the HU group (*p* < 0.05, Figure 3A,C), no significant differences were observed between the HU + IL and HU + IL + HP groups. The phosphorylation of ULK (Ser757) was significantly higher in the HU + IL and HU + IL + HP groups than in the HU group in SOL muscles (*p* < 0.05, Figure 3B) and in the HU + IL + HP group than in the HU and HU + IL groups in GS muscles (*p* < 0.05, Figure 3D).

## 4. Discussion

**Figure 4:**
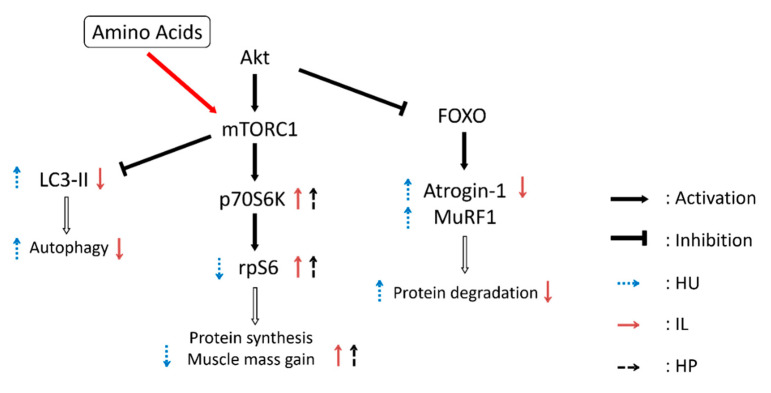
Proposed regulation of the mammalian target of rapamycin complex 1 (mTORC1) signaling pathway induced by intermittent loading (IL) and the administration of high-protein oral nutritional supplement (HP) in rat gastrocnemius muscles during hind limb unloading (HU). FOXO: Forkhead box O.

In this study, we investigated the effects of IL and oral HP administration on mRNA and protein expression levels related to skeletal muscle protein synthesis and breakdown in HU-induced muscle atrophy in rats. IL combined with HP administration was more effective than IL alone in increasing GS muscle mass and phosphorylating targets downstream of the mTORC1 signaling pathway (p70S6K and rpS6). In contrast, SOL muscles did not exhibit significant differences in terms of these results. IL inhibited induction of the ubiquitin-proteasome and autophagy-lysosome systems by HU in both SOL and GS muscles. However, HP administration did not increase these inhibitory effects of IL. HP administration combined with IL was more effective in preventing disuse muscle atrophy in GS muscles. These effects may result from enhanced activation of the mTORC1 signaling pathway (Figure 4).

Phosphorylation of p70S6K and rpS6 represents downstream indicators of mTORC1 signaling pathway activation and is involved in skeletal muscle protein synthesis [12]. Reduced activation of the mTORC1 signaling pathway in disuse muscle atrophy can result in decreased protein synthesis [15,16]. Skeletal muscle atrophy induced by 7, 14, or 28 days of HU reduced mTORC1 and p70S6K phosphorylation in rat SOL muscles [14]. In this study, 14 days of HU significantly reduced rpS6 phosphorylation in SOL and GS muscles. HU decreased relative SOL and GS skeletal muscle masses, which may have resulted from attenuated mTORC1 pathway activation. Miyazaki et al. reported that IL for 4 h per day increased mTORC1 pathway activation during a 7-day period of HU-induced atrophy of rat SOL muscle [17]. In this study, IL for 1 h per day significantly increased p70S6K and rpS6 phosphorylation in GS and SOL muscles. In addition, IL combined with HP administration further increased p70S6K and rpS6 phosphorylation in GS muscles, but not SOL muscles. Skeletal muscle fibers are generally classified as type-I fibers and type-II fibers. Whereas SOL muscles in rats are composed of 83% type-I fibers, GS muscles are composed of 10% type-1 fibers and 90% type-II fibers [35]. p70S6K expression levels were reported to be higher in type-II fibers than in type-I fibers, in both rats and humans [36,37], suggesting that the mTORC1 signaling pathway exerts a greater effect in type-II fibers. Therefore, one reason for the discrepancy in the effects of HP administration between SOL and GS muscles may relate to differences in responsiveness to amino acids between the skeletal muscle fiber types. Specifically, the effects of amino acids may be manifested more readily in GS muscles than in SOL muscles. However, no study has been conducted to examine differences in amino acid responsiveness between different types of skeletal muscle fibers. Changes in the relative SOL and GS skeletal muscle masses showed trends similar to the changes observed in p70S6K and rpS6 phosphorylation. The relative GS muscle masses in rats treated with HU were highest in the group that also underwent IL combined with HP administration.

The muscle-specific E3 ubiquitin ligase atrogin-1 is specifically upregulated during muscle atrophy and promotes muscle protein breakdown [23]. In this study, the mRNA expression levels of atrogin-1 in SOL and GS muscles increased significantly after HU, suggesting that the ubiquitin-proteasome system was induced. IL was previously found to inhibit atrogin-1 expression in SOL muscles in rats treated with HU [17], and our results are consistent with this report. Furthermore, our current results demonstrated that IL reduced atrogin-1-expression levels in GS muscles in a rat model of disuse atrophy. Chronic oral administration of BCAAs attenuated HU-induced atrogin-1 upregulation in SOL muscles [25]. However, in this study, HP administration did not increase the effects of IL in SOL and GS muscles, suggesting that HP administration during IL did not affect the ubiquitin-proteasome system.

Akt phosphorylation serves as an upstream activator of the mTORC1 pathway. Akt phosphorylates FOXO, a transcription factor that promotes atrogin-1 and MuRF1 expression [9,38,39,40]. Phosphorylation of FOXO decreases its activity, after which muscle protein breakdown is inhibited. Notably, IL for 4 h per day has been shown to increase Akt phosphorylation in the SOL muscles of rats with HU-induced muscle atrophy [17]. However, in this study, IL did not induce changes in Akt phosphorylation in SOL muscles. This discrepancy could be explained by differences in the IL intensity or the timing of post-IL skeletal muscle sampling. In addition, Ribeiro et al. reported that leucine administration increased Akt phosphorylation and mTORC1 activity in SOL muscles in a rat model of denervation-induced muscle atrophy [41]. Another report showed that oral BCAA administration did not significantly affect Akt phosphorylation in SOL muscles from rats with HU-induced atrophy [25]. Thus, the effects of administering amino acids on Akt phosphorylation in disuse muscle atrophy have been inconsistent, and the discrepancies have not been fully clarified. The current findings demonstrated that IL combined with HP administration increased Akt phosphorylation in GS muscles. The combination of IL and HP administration may promote activation of the mTORC1 signaling pathway through increased Akt phosphorylation.

The autophagy–lysosome and ubiquitin–proteasome systems play important cellular roles, and LC3 has been described as an indicator of mammalian autophagy [27,42]. In this study, HU increased the LC3B II/I ratio in SOL and GS muscles, indicating that the autophagy-lysosome system was induced by HU. This result is consistent with the studies of SOL and GS muscles reported by Maki et al. and Zhang et al. [25,30]. In contrast, Speacht et al. reported that no significant change occurred in LC3 expression during HU in GS and quadriceps femoris muscles [31]. This discrepancy could be explained by differences in the study designs and the skeletal muscle types. Our results confirmed that IL inhibited the autophagy-lysosome system induced by HU in SOL and GS muscles. However, our data suggested that IL combined with HP administration did not further inhibit the autophagy-lysosome system in SOL or GS muscles. Maki et al. reported that although BCAA administration improved the cross-sectional area of SOL muscles, which was decreased by HU, it did not affect the expression levels of LC3-II following upregulation by HU [25]. Therefore, further studies are necessary to examine the effects of amino acids on disuse atrophy-induced autophagy. Phosphorylation of ULK1, a component of the ULK1 complex, is known to regulate autophagy [28]. Activated mTORC1 phosphorylates ULK1 at Ser757 and then inhibits the autophagy-proteasome system [29,43]. In this study, the combination of IL with HP administration may have altered the phosphorylation of ULK1 at Ser757 in GS muscles. Our results suggest that the decrease in the LC3B II/I ratio and the increase in the inhibitory phosphorylation of ULK1 resulted from mTORC1 activation.

The current study has some limitations. Since no HP-administered group without IL was included in the current study, we could not evaluate the effect of HP itself on HU-induced muscle atrophy. However, in our separate pilot study, HP administration alone, without IL, did not affect the SOL and GS skeletal muscle masses in a rat model of disuse atrophy induced by 14 days of HU (Table S1). Secondly, because the effect of the combination of IL and HP differed between the SOL and GS skeletal muscles, immunostaining for fiber type-specific myosin heavy chain proteins and the measurement of the cross-sectional area of muscles could further assess this difference. In addition, although HP administration combined with IL increased the gastrocnemius muscle mass, measuring the myofiber cross-sectional area is essential to eliminate the possibility that edema or fat infiltration may have affected the obtained skeletal muscle mass. Future studies should be performed to assess whether the combination of IL and protein administration affects other types of muscle atrophy, such as denervation-induced disuse atrophy, cast immobilization-induced disuse atrophy, and age-related muscle atrophy.

## 5. Conclusions

We evaluated the effects of IL and oral administration of HP on skeletal muscle protein synthesis and breakdown in a rat model of HU-induced muscle atrophy. HP administration increased the plasma concentrations of EAAs, BCAAs, and leucine. IL decreased relative SOL and GS muscle mass loss induced by HU, and IL combined with HP administration was more effective in preventing GS muscle atrophy than IL alone. In addition, p70S6K and rpS6 phosphorylation in GS muscles was significantly higher when IL was combined with HP administration, when compared with IL treatment alone. In both SOL and GS muscles, although IL inhibited induction of the ubiquitin-proteasome and autophagy-lysosome systems by HU, HP administration did not increase the effect of IL. The results of this study showed that IL combined with HP administration was more effective in inhibiting skeletal muscle atrophy induced by physical disuse than IL alone in GS muscles. In addition, these findings may also reflect enhanced activation of the mTORC1 signaling pathway. A combination of nutritional and physical strategies may contribute to the development of effective prevention and treatment approaches for disuse muscle atrophy.

## Figures and Tables

**Table 1:** Body weight, caloric intake, and absolute and relative skeletal muscle mass.

	CON	HU	HU + IL	HU + IL + HP
Body weight before HU (g)	221.1 ± 8.6	214.8 ± 7.2	212.4 ± 11.8	215.1 ± 7.2
Body weight after HU (g)	211.4 ± 6.4	186.1 ± 7.2 *	186.9 ± 4.7	190.8 ± 7.7
Caloric intake (kcal/day)	44.3 ± 0.2	43.5 ± 0.4 *	43.4 ± 0.4	43.5 ± 0.4
Soleus muscle (mg)	76.5 ± 3.8	39.3 ± 3.3 *^,a^	51.5 ± 3.2 ^b^	53.0 ± 3.8 ^b^
Soleus muscle/body weight (mg/g)	0.36 ± 0.01	0.21 ± 0.02 *^,a^	0.28 ± 0.02 ^b^	0.28 ± 0.02 ^b^
Gastrocnemius muscle (mg)	957.5 ± 36.4	753.3 ± 41.0 *^,a^	783.2 ± 26.9 ^a^	825.7 ± 41.0 ^b^
Gastrocnemius muscle/body weight (mg/g)	4.5 ± 0.1	4.0 ± 0.1 *^,a^	4.2 ± 0.1 ^b^	4.3 ± 0.1 ^c^

Four groups of rats were assigned: control (CON), hind limb unloading (HU), intermittent loading (IL) during HU (HU + IL), and IL during HU followed by high-protein oral nutritional supplement (HP) administration (HU + IL + HP). The data shown represent the mean ± standard deviation (SD, *n* = 6–10). * *p* < 0.05: compared with CON. ^a, b,^ and ^c^: the different letters going across each row indicate significant differences among the HU, HU + IL, and HU + IL + HP groups as determined using multiple comparison tests (*p* < 0.05).

**Table 2:** Plasma amino acid concentrations.

	CON	HU	HU + IL	HU + IL + HP
EAA (nmol/mL)	1334.5 ± 140.1	1375.7 ± 89.9 ^a^	1366.1 ± 129.5 ^a^	1837.3 ± 200.0 ^b^
BCAA (nmol/mL)	370.1 ± 51.5	452.3 ± 42.1 *^,a^	431.9 ± 72.0 ^a^	818.0 ± 117.3 ^b^
Leucine (nmol/mL)	127.7 ± 20.7	160.5 ± 18.5 *^,a^	155.7 ± 28.7 ^a^	481.0 ± 70.8 ^b^

Four groups of rats were assigned: control (CON), hind limb unloading (HU), intermittent loading (IL) during HU (HU + IL) and IL during HU followed by high-protein oral nutritional supplement (HP) administration (HU + IL + HP). Plasma samples were collected 30 min after the last IL and oral administration. Data are shown as the mean ± SD (*n* = 6–10). * *p* < 0.05: compared with the CON group. ^a^ and ^b^: the different letters going across each row indicate significant differences among the HU, HU + IL, and HU + IL + HP groups as determined using multiple comparison tests (*p* < 0.05). EAA: essential amino acid; BCAA: branched-chain amino acid.

## Supplementary Materials

The following are available online at https://www.mdpi.com/2072-6643/12/9/2729/s1, Table S1: Absolute and relative skeletal muscle mass.

## References

[1] Welle S., Thornton C., Statt M., McHenry B. (1994). Postprandial myofibrillar and whole body protein synthesis in young and old human subjects. Am. J. Physiol..

[2] Yoshizawa F., Kimball S.R., Vary T.C., Jefferson L.S. (1998). Effect of dietary protein on translation initiation in rat skeletal muscle and liver. Am. J. Physiol..

[3] Yarasheski K.E., Zachwieja J.J., Bier D.M. (1993). Acute effects of resistance exercise on muscle protein synthesis rate in young and elderly men and women. Am. J. Physiol..

[4] Volpi E., Kobayashi H., Sheffield-Moore M., Mittendorfer B., Wolfe R.R. (2003). Essential amino acids are primarily responsible for the amino acid stimulation of muscle protein anabolism in healthy elderly adults. Am. J. Clin. Nutr..

[5] Anthony J.C., Yoshizawa F., Anthony T.G., Vary T.C., Jefferson L.S., Kimball S.R. (2000). Leucine stimulates translation initiation in skeletal muscle of postabsorptive rats via a rapamycin-sensitive pathway. J. Nutr..

[6] Fujita S., Volpi E. (2006). Amino acids and muscle loss with aging. J. Nutr..

[7] Foletta V.C., White L.J., Larsen A.E., Léger B., Russell A.P. (2011). The role and regulation of MAFbx/atrogin-1 and MuRF1 in skeletal muscle atrophy. Pflug. Arch..

[8] Jackman R.W., Kandarian S.C. (2004). The molecular basis of skeletal muscle atrophy. Am. J. Physiol. Cell Physiol..

[9] Bonaldo P., Sandri M. (2013). Cellular and molecular mechanisms of muscle atrophy. Dis. Model. Mech..

[10] Stein T.P., Wade C.E. (2005). Metabolic consequences of muscle disuse atrophy. J. Nutr..

[11] Drummond M.J., Dreyer H.C., Fry C.S., Glynn E.L., Rasmussen B.B. (2009). Nutritional and contractile regulation of human skeletal muscle protein synthesis and mTORC1 signaling. J. Appl. Physiol..

[12] Makanae Y., Fujita S. (2015). Role of exercise and nutrition in the prevention of sarcopenia. J. Nutr. Sci. Vitaminol..

[13] Li F., Yin Y., Tan B., Kong X., Wu G. (2011). Leucine nutrition in animals and humans: mTOR signaling and beyond. Amino Acids..

[14] Dupont E., Cieniewski-Bernard C., Bastide B., Stevens L., Dupont E., Cieniewski-Bernard C., Bastide B., Stevens L. (2011). Electrostimulation during hindlimb unloading modulates PI3K-AKT downstream targets without preventing soleus atrophy and restores slow phenotype through ERK. Am. J. Physiol. Regul. Integr. Comp. Physiol..

[15] Bodine S.C., Stitt T.N., Gonzalez M., Kline W.O., Stover G.L., Bauerlein R., Zlotchenko E., Scrimgeour A., Lawrence J.C., Glass D.J. (2001). Akt/mTOR pathway is a crucial regulator of skeletal muscle hypertrophy and can prevent muscle atrophy in vivo. Nat. Cell Biol..

[16] Hornberger T.A., Hunter R.B., Kandarian S.C., Esser K.A. (2001). Regulation of translation factors during hindlimb unloading and denervation of skeletal muscle in rats. Am. J. Physiol. Cell Physiol..

[17] Miyazaki M., Noguchi M., Takemasa T. (2008). Intermittent reloading attenuates muscle atrophy through modulating Akt/mTOR pathway. Med. Sci. Sports Exerc..

[18] Sandri M. (2013). Protein breakdown in muscle wasting: Role of autophagy-lysosome and ubiquitin-proteasome. Int. J. Biochem. Cell Biol..

[19] Ikemoto M., Nikawa T., Takeda S., Watanabe C., Kitano T., Baldwin K.M., Izumi R., Nonaka I., Towatari T., Teshima S. (2001). Space shuttle flight (STS-90) enhances degradation of rat myosin heavy chain in association with activation of ubiquitin-proteasome pathway. FASEB J..

[20] Taillandier D., Aurousseau E., Meynial-Denis D., Bechet D., Ferrara M., Cottin P., Ducastaing A., Bigard X., Guezennec C.Y., Schmid H.P. (1996). Coordinate activation of lysosomal, Ca^2+^-activated and ATP-ubiquitin-dependent proteinases in the unweighted rat soleus muscle. Biochem. J..

[21] Tawa N.E., Odessey R., Goldberg A.L. (1997). Inhibitors of the proteasome reduce the accelerated proteolysis in atrophying rat skeletal muscles. J. Clin. Investig..

[22] Bodine S.C., Latres E., Baumhueter S., Lai V.K., Nunez L., Clarke B.A., Poueymirou W.T., Panaro F.J., Na E., Dharmarajan K. (2001). Identification of ubiquitin ligases required for skeletal muscle atrophy. Science.

[23] Bodine S.C., Baehr L.M. (2014). Skeletal muscle atrophy and the E3 ubiquitin ligases MuRF1 and MAFbx/atrogin-1. Am. J. Physiol. Endocrinol. Metab..

[24] Jang J., Yun H.Y., Park J., Lim K. (2015). Protective effect of branched chain amino acids on hindlimb suspension-induced muscle atrophy in growing rats. J. Exerc. Nutr. Biochem..

[25] Maki T., Yamamoto D., Nakanishi S., Iida K., Iguchi G., Takahashi Y., Kaji H., Chihara K., Okimura Y. (2012). Branched-chain amino acids reduce hindlimb suspension-induced muscle atrophy and protein levels of atrogin-1 and MuRF1 in rats. Nutr. Res..

[26] Zhao J., Brault J.J., Schild A., Cao P., Sandri M., Schiaffino S., Lecker S.H., Goldberg A.L. (2007). FoxO3 coordinately activates protein degradation by the autophagic/lysosomal and proteasomal pathways in atrophying muscle cells. Cell Metab..

[27] Glick D., Barth S., Macleod K.F. (2010). Autophagy: Cellular and molecular mechanisms. J. Pathol..

[28] Ganley I.G., Lam D.H., Wang J., Ding X., Chen S., Jiang X. (2009). ULK1.ATG13.FIP200 complex mediates mTOR signaling and is essential for autophagy. J. Biol. Chem..

[29] Kim J., Kundu M., Viollet B., Guan K.L. (2011). AMPK and mTOR regulate autophagy through direct phosphorylation of Ulk1. Nat. Cell Biol..

[30] Zhang S.F., Zhang Y., Li B., Chen N. (2018). Physical inactivity induces the atrophy of skeletal muscle of rats through activating AMPK/FoxO3 signal pathway. Eur. Rev. Med. Pharmacol..

[31] Speacht T.L., Krause A.R., Steiner J.L., Lang C.H., Donahue H.J. (2018). Combination of hindlimb suspension and immobilization by casting exaggerates sarcopenia by stimulating autophagy but does not worsen osteopenia. Bone.

[32] Morey E.R., Sabelman E.E., Turner R.T., Baylink D.J. (1979). A new rat model simulating some aspects of space flight. Physiologist.

[33] Kido K., Sato K., Makanae Y., Ato S., Hayashi T., Fujita S. (2016). Herbal supplement Kamishimotsuto augments resistance exercise-induced mTORC1 signaling in rat skeletal muscle. Nutrition.

[34] Livak K.J., Schmittgen T.D. (2001). Analysis of relative gene expression data using relative gene expression using real-time quantitative PCR and the 2^−ΔΔ*C*t^ Method. Methods.

[35] Bär A., Pette D. (1988). Three fast myosin heavy chains in adult rat skeletal muscle. FEBS Lett..

[36] Atherton P.J., Higginson J.M., Singh J., Wackerhage H. (2004). Concentrations of signal transduction proteins exercise and insulin responses in rat extensor digitorum longus and soleus muscles. Mol. Cell. Biochem..

[37] Edman S., Söderlund K., Moberg M., Apró W., Blomstrand E. (2019). mTORC1 signaling in individual human muscle fibers following resistance exercise in combination with intake of essential amino acids. Front. Nutr..

[38] Stitt T.N., Drujan D., Clarke B.A., Panaro F., Timofeyva Y., Kline W.O., Gonzalez M., Yancopoulos G.D., Glass D.J. (2004). The IGF-1/PI3K/Akt pathway prevents expression of muscle atrophy-induced ubiquitin ligases by inhibiting FOXO transcription factors. Mol. Cell..

[39] Sandri M., Sandri C., Gilbert A., Skurk C., Calabria E., Picard A., Walsh K., Schiaffino S., Lecker S.H., Goldberg A.L. (2004). Foxo transcription factors induce the atrophy-related ubiquitin ligase atrogin-1 and cause skeletal muscle atrophy. Cell.

[40] Brunet A., Bonni A., Zigmond M.J., Lin M.Z., Juo P., Hu L.S., Anderson M.J., Arden K.C., Blenis J., Greenberg M.E. (1999). Akt promotes cell survival by phosphorylating and inhibiting a Forkhead transcription factor. Cell.

[41] Ribeiro C.B., Christofoletti D.C., Pezolato V.A., de Cássia Marqueti Durigan R., Prestes J., Tibana R.A., Pereira E.C., de Sousa Neto I.V., Durigan J.L., da Silva C.A. (2015). Leucine minimizes denervation-induced skeletal muscle atrophy of rats through akt/mtor signaling pathways. Front. Physiol..

[42] Kabeya Y., Mizushima N., Ueno T., Yamamoto A., Kirisako T., Noda T., Kominami E., Ohsumi Y., Yoshimori T. (2000). LC3, a mammalian homologue of yeast Apg8p, is localized in autophagosome membranes after processing. EMBO J..

[43] Egan D.F., Shackelford D.B., Mihaylova M.M., Gelino S., Kohnz R.A., Mair W., Vasquez D.S., Joshi A., Gwinn D.M., Taylor R. (2011). Phosphorylation of ULK1 (hATG1) by AMP-activated protein kinase connects energy sensing to mitophagy. Science.

