# Supplementary material for: Protein Supplementation Enhances the Effects of Intermittent Loading on Skeletal Muscles by Activating the mTORC1 Signaling Pathway in a Rat Model of Disuse Atrophy
Source: Nutrients. 2020 Sep 7;12(9):2729. doi: 10.3390/nu12092729 (PMC7551819; doi:10.3390/nu12092729)
Supplement: Supplementary file 1 [file nutrients-12-02729-s001.pdf]

Supplementary Materials

Table 1. Absolute and relative skeletal muscle mass.

	CON	HU	HU+HP
Soleus muscle (mg)	78.7 ± 4.1	41.8 ± 2.5 *	43.8 ± 0.9
Soleus muscle/body weight (mg/g)	0.38 ± 0.02	0.22 ± 0.01 *	0.23 ± 0.01
Gastrocnemius muscle (mg)	950.8 ± 29.0	758.8 ± 30.2 *	785.0 ± 34.9
Gastrocnemius muscle/body weight (mg/g)	4.6 ± 0.1	4.1 ± 0.2 *	4.1 ± 0.1

Three groups of F344/DuCrlCrlj rats were assigned: control (CON), 14 days of hindlimb unloading (HU), and high-protein oral nutritional supplement (HP) administration during HU (HU + HP). The data shown represent the mean ± SD (n = 5–10). *$p < 0.05$: compared with CON.
